# Sustaining Identity Through Spirituality In End-of-Life Dementia Care: An Analytic Auto-ethnography

**DOI:** 10.1093/geroni/igaf122.3132

**Published:** 2025-12-31

**Authors:** Eunkyung Kim, Soobin Park, Sooyoung Park

**Affiliations:** Midwest University, Wentzville, Missouri, United States; Washington University in St. Louis, St. Louis, Missouri, United States; Samsung Medical Center, Gangnam-gu, Seoul, Republic of Korea

## Abstract

Spirituality, originating from Latin meaning “breath of life,” transcends intellectual capacity, deeply defining who we are. Yet, spirituality remains one of the most neglected dimensions in end-of-life dementia care. Although spiritual support for persons living with dementia (PLWD) is recognized as important, it often remains unaddressed because care providers find it challenging to identify or apply spiritual needs, particularly during advanced dementia. Consequently, spiritual needs frequently go undocumented in care plans and discussions. Additionally, limited empirical research on the spiritual needs of PLWD contributes to uncertainty about integrating spirituality into dementia care. Using analytic auto-ethnography, which enables researchers to utilize personal experiences to generate specific, contextually relevant understanding, this study draws upon my perspectives as an Asian woman, former caregiver to my husband diagnosed with dementia, and current social work scholar. Reflecting on over a decade of caregiving through reflexive approach of archival materials (memoirs, photographs) and concurrent self-observations (diaries, audio-visual recordings), key themes emerged highlighting the nuanced nature of spiritual caregiving. Findings revealed spirituality as highly individualized, extending beyond religious rituals and deeply connected to life history, identity, and meaningful experiences. Assessing spiritual needs involves actively recalling significant past conversations and preferences through dialogue with family and friends. For example, attending annual academic conferences with my husband, a former professor, exemplified spiritual care rooted in personal history. The study implies that recognizing and including individualized spiritual practices restores dignity and personhood, guiding care providers toward spiritually competent and person-centered dementia care, thus addressing key gaps in practice and research.

